# Prevalence and long-term outcomes of NAFLD and cardiovascular-kidney-metabolic health in the United States

**DOI:** 10.1016/j.ajpc.2025.101049

**Published:** 2025-06-18

**Authors:** Jian Liu, Lei Gao, Junlong Chen, Zhenghao Li, Wenhang Zhao, Shiwei Qin, Xuesong Wen, Dongying Zhang

**Affiliations:** a Department of Cardiology, Chongqing Emergency Medical Center, Chongqing University Central Hospital, School of Medicine, Chongqing University, Chongqing, PR China; b The First College of Clinical Medicine, Chongqing Medical University, Chongqing, PR China; c Department of Cardiology, The First Affiliated Hospital of Chongqing Medical University, Chongqing, PR China; d Department of Cardiothoracic Surgery, The First Affiliated Hospital of Chongqing Medical University, Chongqing, PR China

**Keywords:** CKM syndrome, NAFLD, Mortality, Comorbidity, NHANES III

## Abstract

**Background and aims:**

To explore non-alcoholic fatty liver disease (NAFLD)’s role within the novel cardiovascular-kidney-metabolic (CKM) syndrome framework, given their overlapping pathophysiological mechanisms.

**Methods:**

We analyzed 10,985 U.S. adults from the National Health and Nutrition Examination Survey III (1988–1994), with mortality follow-up until 2019. NAFLD was assessed via ultrasonography, and liver fibrosis was determined by the NAFLD fibrosis score and Fibrosis-4 index. CKM stages and predicted cardiovascular (CVD) event risk were assessed per American Heart Association guidelines.

**Results:**

Age-standardized prevalence of NAFLD progressively increased across CKM stages, from 21.8 % at stage 0, 24.1 % at stage 1, 40.0 % at stage 2, and 40.2 % at advanced CKM. NAFLD was significantly associated with CKM progression (odds ratio = 2.01; 95 % confidence interval: 1.84–2.20) in ordinal logistic regression. During a median follow-up of 27.9 years, 3,772 deaths occurred. NAFLD was associated with a 26 % increased risk of all-cause mortality in multivariable Cox models; however, the association became non-significant after adjustment for specific components of CKM. Among participants with NAFLD, mortality risk increased stepwise with advancing CKM. Predicted CVD event risk demonstrated superior prognostic value than NAFLD fibrosis score and Fibrosis-4 score (area under the curve = 0.88). CKM stage significantly mediated the association between NAFLD and mortality, with a mediation proportion of 35.4 %.

**Conclusions:**

NAFLD exhibits high comorbidity with CKM syndrome, and the high prevalence of NAFLD at CKM 0 highlights early metabolic risk. NAFLD is not an independent mortality predictor but accelerates CKM progression to increased mortality.

## Graphical abstract

Abbreviation: NAFLD, Nonalcoholic fatty liver disease; CKM, Cardiovascular-Kidney-Metabolic syndrome; AHA, American Heart Association; NHANES, National Health and Nutrition Examination Survey; OR, odds ratio; CI, confidence interval; HR, hazard ratio; FIB-4, fibrosis-4 index; NFS, NAFLD fibrosis score; AUC, area under the curve.

**Figure:**
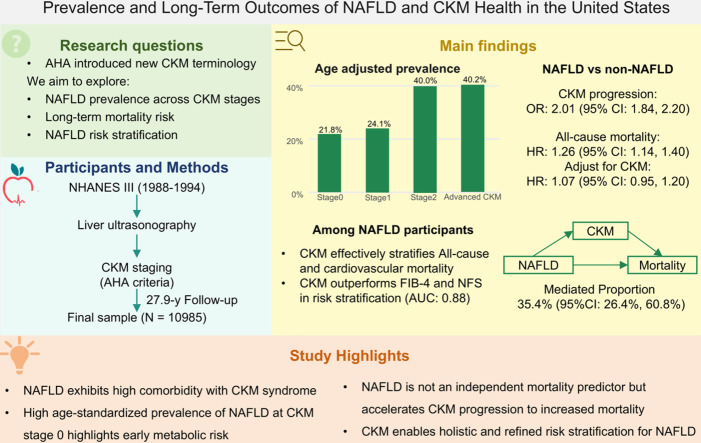

## Glossary

CKD: Chronic kidney disease
CKM: cardiovascular-kidney-metabolic syndrome;
CVD: cardiovascular disease
FIB-4: Fibrosis-4 score
HEI: Healthy Eating Index
HOMA-IR: Homeostasis model assessment of insulin resistance
MAFLD: Metabolic-associated fatty liver disease
MASLD: Metabolic dysfunction–associated steatotic liver disease
MetS: Metabolic syndrome
NAFLD: Non-alcoholic fatty liver disease
NFS: NAFLD fibrosis score
NHANES: National Health and Nutrition Examination Survey
PIR: Poverty income ratio
PREVENT: Predicting Risk of cardiovascular disease EVENTs
SLD: Steatosis liver disease

## 1 Introduction

The cardiovascular-renal-metabolic (CKM) syndrome is a newly defined health disorder, as outlined in the 2023 consensus statement from the American Heart Association [1]. This syndrome is characterized by the pathophysiological interactions among obesity, diabetes, chronic kidney disease (CKD), and cardiovascular disease (CVD), and imposes profound impacts on morbidity and mortality [2]. Recent studies have highlighted the high prevalence of CKM syndrome among American adults, with nearly 90 % meeting the criteria for stage 1 or higher between 2011 and 2020. Notably, 15 % of American adults were classified as having advanced CKM stages (stage 3-4) [3]. Given its systemic nature, CKM is associated with a substantial increase in the risk of CVD events, kidney function decline, and all-cause mortality [4,5].

Non-alcoholic fatty liver disease (NAFLD), recently redefined as metabolic dysfunction–associated steatotic liver disease (MASLD), is one of the most common metabolic disorders worldwide [6,7]. NAFLD is closely linked to insulin resistance, oxidative stress, vascular dysfunction, and chronic inflammation, all of which are critical contributors to CKM syndrome [[2], [7], [8]]. Previous studies have shown that NAFLD is closely associated with an increased risk of CVD and CKD, but its precise role within the CKM framework remains poorly defined. It is unclear whether NAFLD primarily serves as an initiator of CKM progression or acts synergistically with other metabolic disorders to exacerbate multi-organ dysfunction. Currently, NAFLD risk stratification is primarily based on coexisting metabolic risk factors, non-invasive liver fibrosis assessment tools such as the fibrosis-4 (FIB-4) index and the NAFLD fibrosis score (NFS), or liver stiffness measurement via vibration-controlled transient elastography [9], whether the CKM framework offers a more comprehensive and effective approach for NAFLD risk stratification remains unclear. Despite the growing recognition of the interconnections between NAFLD, type 2 diabetes, CVD, and CKD, most existing studies have focused on the outcomes of single diseases [10,11], without considering their coexistence and mutual influence. This reductionist approach may underestimate the broader impact of NAFLD on systemic metabolic health. Addressing these knowledge gaps is crucial to refining NAFLD risk stratification and improving targeted intervention strategies. Given the high prevalence of hepatic steatosis in non-obese individuals and those without overt metabolic dysfunction, who may nonetheless experience adverse outcomes, we adopted the term NAFLD in this study to avoid excluding such at-risk populations [12,13]. Although the newer terms MASLD offer stronger alignment with metabolic dysfunction and adverse cardiometabolic profiles, relying solely on these definitions may inadvertently miss individuals with metabolically “silent” steatosis who are at earlier stages of disease but may still carry significant long-term risk. This choice ensures a more inclusive assessment of hepatic steatosis and its broader relevance within the CKM framework.

In this study, we used a nationally representative sample of U.S. adults to investigate the association between NAFLD and CKM progression, as well as its impact on all-cause and cardiovascular mortality, offering new insights into the role of NAFLD within the CKM framework.

## 2 Materials and methods

### 2.1 Study population

This study utilized data from the National Health and Nutrition Examination Survey III (NHANES III, 1988–1994), a nationally representative survey designed to assess the health and nutritional status of non-institutionalized US civilians [12]. All participants, or their guardians, provided written informed consent prior to data collection. This secondary analysis of de-identified data was exempt from review and adhered to the Strengthening the Reporting of Observational Studies in Epidemiology [STROBE] guidelines for observational studies.

### 2.2 Data collection

The demographic data of the participants, along with their physical examinations, laboratory tests, lifestyle and dietary habits, medical conditions, electrocardiogram, and hepatic and gallbladder ultrasound data, were meticulously collected. Demographic information encompassed age, gender, race and ethnicity, education level, and poverty income ratio (PIR). The income levels were categorized into low (PIR < 1.3), medium (PIR 1.3 - 3.5), and high (PIR > 3.5) [13]. The physical examination included body mass index (BMI), waist circumference, and blood pressure (average of three measurements). Laboratory test results comprised triglycerides, total cholesterol, high-density lipoprotein cholesterol (HDL-C), fasting blood glucose, glycated hemoglobin A1c (HbA1c), insulin, estimated glomerular filtration rate (eGFR), urinary albumin to creatinine ratio (UACR), C-reactive protein (CRP), platelet count, albumin, alanine aminotransferase (ALT), and aspartate aminotransferase (AST). Insulin resistance was determined as the homeostasis model assessment of insulin resistance (HOMA-IR) score ≥ 2.5. Given the lack of universally accepted cutoff values for defining insulin resistance using HOMA-IR, we applied alternative thresholds of HOMA-IR ≥ 2.0 and ≥ 1.0 to represent varying degrees of insulin resistance. Lifestyle and dietary data included smoking status, alcohol consumption, and the Healthy Eating Index (HEI). The HEI-2010 (0-100) was calculated from two 24-hour dietary recalls using the simple HEI scoring algorithm developed by the National Institutes of Health (NIH), with healthy eating defined as an HEI score of ≥ 69.3 [14]. Detailed measurement procedures for all variables are publicly accessible on the website (www.cdc.gov/nchs/nhanes/).

### 2.3 Definition of NAFLD and advanced fibrosis

Hepatic ultrasound examinations were originally performed on NHANES III participants aged 20 to 74 years using Toshiba Sonolayer SSA-90A scanners. Between 2009 and 2010, archived gallbladder ultrasound videotapes were re-evaluated to assess hepatic steatosis. Three trained ultrasound reviewers, under the supervision of a board-certified radiologist specialized in hepatic imaging, conducted the assessments using a standardized algorithm based on five imaging features: (1) parenchymal brightness, (2) liver-to-kidney contrast, (3) deep beam attenuation, (4) bright vessel walls, and (5) gallbladder wall definition. The liver was graded as having normal, mild, moderate, or severe steatosis based on the combined presence of these features. For further details on methodology and quality control, please refer to other chapters [15]. Here, we use the term NAFLD to remain consistent with the NHANES III dataset and its historical definitions, which predate the adoption of the MASLD terminology. The diagnosis of NAFLD encompasses two sets of criteria: mild-to-severe or moderate-to-severe hepatic steatosis, in the absence of other chronic liver diseases or excessive alcohol consumption (< 20 g/day for males and < 10 g/day for females) [16,17]. Moderate-to-severe hepatic steatosis was used as the main analysis in the subsequent analysis. Regarding liver fibrosis, we calculated the NFS and FIB-4 scores based on published equations [18,19]. NFS = -1.675 + 0.037 × age (years) + 0.094 × BMI (kg/m2) + 1.13 × impaired fasting glucose/diabetes (yes = 1, no = 0) + 0.99 × AST/ALT ratio - 0.013 × platelet (×10^9^/L) - 0.66 × albumin (g/dL). FIB-4 score = (Age [year] × AST [U/L]) / (platelets [10^9^/L] × (ALT [U/L])^1/2^). Advanced liver fibrosis was defined as NFS > 0.676 or FIB-4 score > 2.67.

### 2.4 Definition of CKM syndrome

Participants were classified into CKM stages according to the AHA’s 2023 framework. Stage 0 represents the absence of CKM risk factors (i.e., no excess/dysfunctional adiposity, metabolic abnormalities, or CKD). Stage 1 indicates the presence of excessive or dysfunctional adiposity, operationalized as overweight or obesity (BMI ≥25 kg/m²), abdominal obesity (waist circumference ≥102 cm in men or ≥88 cm in women), or glycemic dysregulation (fasting glucose 100–124 mg/dL or HbA1c 5.7–6.4 %) without other CKM abnormalities. Stage 2 includes individuals with established metabolic risk factors (e.g., hypertension, diabetes, dyslipidemia) or moderate-to-high risk CKD. Stage 3 reflects subclinical CVD, defined using AHA-recommended risk equivalents due to the lack of imaging or biomarker data in NHANES III. Specifically, subclinical CVD was defined as either very high-risk CKD or a predicted 10-year CVD risk ≥20 % based on the PREVENT equation (full model via the preventr R package) [20]. Stage 4 denotes established clinical CVD, including self-reported history of coronary heart disease, myocardial infarction, heart failure, or stroke. CKD classification follows the Kidney Disease Improving Global Outcomes (KDIGO) guidelines, and eGFR was calculated using the 2021 CKD-EPI creatinine equation without race adjustment [21]. More detailed staging criteria are provided in Supplementary Table S1–S2.

### 2.5 Ascertainment of mortality

Mortality status was determined via probabilistic record linkage with the National Death Index. Survival time was calculated from the completion date of the NHANES III survey to the date of death or December 31, 2019 (the censoring date), whichever occurred first. All-cause mortality was defined as death from any cause, and cardiovascular mortality was defined as death attributable to diseases of the heart and cerebrovascular diseases.

## 3 Statistical analysis

Following the sampling design employed in NHANES III, we utilized the appropriate sampling weights to analyze the data, ensuring that it accurately represents the national population of the entire United States. To address missing data, multiple imputation was performed to generate five imputed datasets, with one randomly selected dataset used for the primary analysis. Baseline differences between subjects with missing data and those with complete data were presented in Supplementary Table S3. Baseline characteristics were compared by CKM and NAFLD status. Continuous variables were presented as weighted means with standard errors (SE), while categorical variables were expressed as unweighted frequencies with weighted percentages. Group comparisons were conducted using the design-based Kruskal-Wallis test and Rao-Scott chi-square test as appropriate. Due to limitations in NHANES III, including the absence of peripheral artery disease and atrial fibrillation diagnoses for clinical CVD staging, and the use of risk equivalents to define subclinical CVD, stages 3 and 4 may be subject to misclassification. Therefore, we combined them into an “advanced CKM” group in the main analyses [22,23]. Age-standardized prevalence and mortality were calculated using the direct method based on the 2000 U.S. Census data for the age groups 20–39, 40–59, and 60–74.

Ordinal logistic regression was used to examine the association between NAFLD and CKM stage or CKD risk progression. Binary logistic regression was performed to evaluate the relationship between CKM stage progression and advanced liver fibrosis. To validate the role of the CKM framework in NAFLD risk stratification, Cox proportional hazard models were employed to assess the association between CKM status and mortality in NAFLD patients, with hazard ratios (HRs) and 95 % confidence intervals (CIs) reported. We assessed the proportional hazards assumption using the Kaplan-Meier curve and Schoenfeld residuals and found that it was satisfied for the CKM stage and all other covariates. Receiver operating characteristic curve (ROC) analysis was performed to compare the predictive performance of NFS, FIB-4, and Predicted 10-year CVD risk for mortality in NAFLD patients. Then multivariable Cox proportional model was used to investigate the independent association of NAFLD with mortality after consideration for potential demographic and clinical confounders. Regression-based mediation analyses were conducted to quantify the total effect, natural indirect effects, and natural direct effects of CKM status in the association between NAFLD and all-cause mortality.

To ensure the robustness of the findings, sensitivity analyses were conducted. First, subgroup analyses were performed, with interaction terms included in the models to assess potential effect modification. Interaction p-values were calculated using likelihood ratio tests. Second, Fine and Gray's subdistribution hazards regression was applied to assess the association between CKM status and cardiovascular mortality, considering non-cardiovascular death as a competing event. Third, complete-case analysis was conducted using the dataset without imputation. Fourth, the analysis was repeated using two alternative NAFLD definitions: one including mild-to-severe hepatic steatosis and another restricted to moderate-to-severe hepatic steatosis as the primary analysis.

All statistical analyses were conducted using R (version 4.3.1). Survey-adjusted analyses were performed using the survey package. Multiple imputation, mediation analyses, and competing risk analyses were conducted using the mice, CMAverse, and cmprsk packages, respectively, without applying NHANES survey weights due to software limitations. A two-tailed P < 0.05 was considered statistically significant.

## 4 Results

Out of 13856 adults who underwent hepatic and gallbladder ultrasonography at mobile examination centers, 1415 individuals were excluded due to incomplete mortality status, missing alcohol consumption, or positive viral hepatitis serology. 1318 participants were excluded for missing values required for CKM staging. In addition, 112 cases had missing data for non-invasive liver fibrosis assessment, and 64 individuals with missing insulin or CRP measurements were excluded, leading to the final sample of 10985 participants (Supplementary Fig. S1). Supplementary Table S4 presents a comparison between included and excluded individuals, revealing modest differences in demographic and lifestyle factors.

### 4.1 Demographic and clinical characteristics by CKM and NAFLD status

**Fig. 1:**
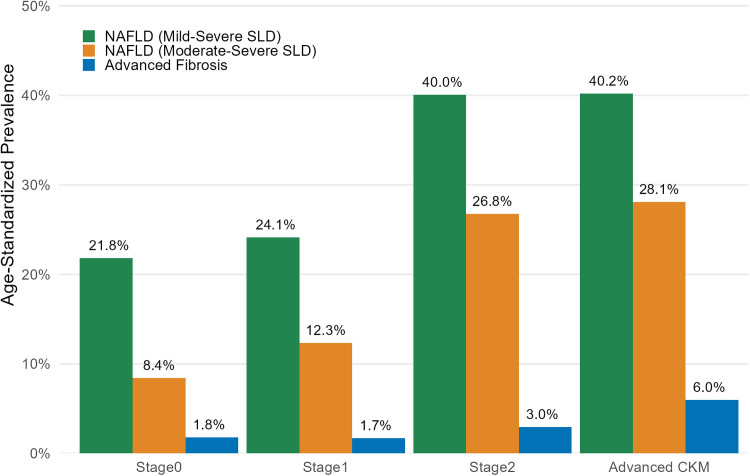
Age-Standardized Prevalence of NAFLD and Advanced Liver Fibrosis Across Cardiovascular-Kidney-Metabolic (CKM) Syndrome Stages. Abbreviation: NAFLD, Nonalcoholic fatty liver disease; SLD, steatosis liver disease.

Among the 10985 eligible participants, the mean age was 43.8 years, 47.7 % were male, 75.0 % were White, 8.4 % were Black, and 7.8 % were Mexican-American. A total of 7.8 % of the participants met the criteria for CKM Stage 0, 7.5 % for Stage 1, 66.4 % for Stage 2, 4.5 % for Stage 3, and 13.8 % for Stage 4 (Table 1). Compared to participants with CKM Stages 0–2, those with advanced CKM (Stages 3–4) were older, more likely to be male, and had lower education and income levels. Additionally, they exhibited significantly higher CRP levels (0.07 mg/dL vs. 0.01–0.04 mg/dL, P < 0.001), and were more likely to be insulin resistance. The prevalence of NAFLD and liver fibrosis also increased progressively across CKM stages. Among participants with advanced CKM, 30.8 % had NAFLD and 11.5 % had advanced liver fibrosis (Table 1). Fig. 1 illustrated age-standardized prevalence patterns of NAFLD and advanced liver fibrosis across CKM stages. NAFLD prevalence increased steeply in early CKM progression, rising from 8.4 % (Stage 0) to 26.8 % (Stage 2), but plateaued in advanced CKM (Stage 2: 26.8 % vs. advanced CKM: 28.1 %). In contrast, advanced liver fibrosis followed a different trajectory, with a low prevalence in CKM Stages 0 and 1 (1.8 % and 1.7 %, respectively), a modest increase in Stage 2 (3.0 %), and a marked rise in advanced CKM (6.0 %).

**Table 1:** Baseline characteristics of the study population, stratified by Cardiovascular-Kidney-Metabolic health status.

Characteristic	Cardiovascular-Kidney-Metabolic syndrome stage							
	Stage 0 N = 1751 (7.8)	Stage 1 N = 1740 (7.5)	Stage 2 N = 5860 (66.4)	Stage 3 N = 455 (4.5)	Stage 4 N = 1176 (13.8)	P-value	Advanced CKM N = 1613 (18.3)	P-value
Age, years	32.95 (0.35)	37.28 (0.47)	43.98 (0.37)	66.90 (0.55)	58.15 (0.52)	<0.001	60.14 (0.45)	<0.001
Age group ( %)						<0.001		<0.001
20-39	1423 (76.4)	1159 (62.9)	2419 (42.1)	9 (1.43)	46 (3.82)		55 (3.3)	
40-59	275 (21.0)	469 (31.2)	2140 (40.2)	43 (12.0)	436 (44.9)		479 (37.4)	
60-74	56 (2.57)	112.0 (5.87)	1,301 (17.7)	403 (86.5)	694 (51.3)		1097 (59.3)	
Male ( %)	641 (34.9)	707 (45.1)	2,869 (52.4)	260 (53.7)	615 (52.1)	<0.001	875 (52.4)	<0.001
Race/ethnicity ( %)						<0.001		<0.001
Mexican-American	506 (4.6)	558 (6.7)	1885 (5.8)	93 (2.6)	288 (3.6)		381 (3.4)	
Non-Hispanic black	419 (7.3)	583 (13.6)	1532 (9.5)	154 (14.8)	297 (9.9)		451 (11.1)	
Non-Hispanic white	741 (79.8)	525 (71.4)	2206 (76.8)	194 (74.7)	545 (79.1)		739 (78.1)	
Other	88 (8.1)	74 (8.3)	237 (7.8)	14 (7.8)	46 (7.3)		60 (7.4)	
Smoking status ( %)						<0.001		<0.001
Never	1021 (52.3)	969 (48.9)	2843 (44.5)	173 (32.6)	404 (29.4)		577 (30.1)	
Former	238 (17.2)	316 (22.7)	1,477 (27.6)	143 (34.8)	443 (40.5)		586 (39.2)	
Current	495 (30.5)	455 (28.4)	1,540 (27.9)	139 (32.6)	329 (30.2)		468 (30.7)	
Less than high school ( %)	427 (14.7)	557 (18.2)	2,219 (23.0)	254 (41.0)	600 (36.4)	<0.001	854 (37.4)	<0.001
Married ( %)	878 (56.6)	987 (63.3)	3750 (67.9)	289 (63.0)	774 (69.3)	<0.001	1063 (67.9)	<0.001
Low income ( %)	515 (16.7)	584 (17.4)	1877 (17.2)	145 (22.8)	385 (20.6)	0.093	530 (21.1)	0.081
Healthy eating ( %)	552 (34.0)	479 (29.1)	1942 (35.1)	182 (42.1)	423 (38.3)	<0.001	605 (39.2)	<0.001
C-reactive protein, mg/dL	0.27 (0.01)	0.34 (0.01)	0.40 (0.01)	0.63 (0.04)	0.60 (0.04)	<0.001	0.61 (0.03)	<0.001
Insulin resistance ( %)								
HOMA-IR≥2.5	113 (4.10)	540 (22.9)	2825 (40.0)	323 (74.4)	660 (48.4)	<0.001	983 (54.3)	<0.001
HOMA-IR≥2.0	251 (10.5)	814 (39.0)	3,663 (55.6)	366 (82.1)	806 (61.2)	<0.001	1172 (65.9)	<0.001
HOMA-IR≥1.0	1308 (71.8)	1620 (91.3)	5532 (92.9)	444 (98.0)	1108 (92.6)	<0.001	1552 (93.8)	<0.001
NAFLD (Mild-Severe SLD, %)	388 (21.9)	408 (20.9)	2346 (36.7)	208 (48.3)	540 (43.6)	<0.001	748 (44.7)	<0.001
NAFLD (Moderate-Severe SLD, %)	146 (7.3)	207 (9.6)	1573 (24.3)	146 (33.3)	374 (29.9)	<0.001	520 (30.6)	<0.001
Advanced liver fibrosis ( %)	11 (0.5)	16 (0.9)	204 (2.6)	93 (17.9)	137 (9.7)	<0.001	230 (11.5)	<0.001
All-cause death ( %)	182 (8.9)	256 (14.7)	2033 (30.7)	422 (94.5)	879 (71.3)	<0.001	1301 (76.6)	<0.001
Cardiovascular death ( %)	33 (1.3)	52 (2.8)	628 (8.6)	169 (41.4)	367 (27.9)	<0.001	536 (40.0)	<0.001

Abbreviation: CKD, chronic kidney disease; NAFLD, Nonalcoholic fatty liver disease; SLD, steatosis liver disease; CKM, Cardiovascular-Kidney-Metabolic syndrome. Continuous variables are presented as weighted means with standard errors, while categorical variables are presented as unweighted frequencies with weighted percentages.

Compared to those without NAFLD, individuals with NAFLD (mild-to-severe SLD) were older, more likely to be Mexican-American, and had lower levels of education. They also exhibited a greater prevalence of metabolic abnormalities, including elevated C-reactive protein (0.44 vs. 0.36 mg/dL, P < 0.001), insulin resistance (HOMA-IR >2.5: 51.6 % vs. 22.3 %, P < 0.001), and multiple components of CKM syndrome, such as obesity, central obesity, hypertension, diabetes, and metabolic syndrome (Table 2). Additionally, NAFLD was associated with a higher CKD risk category (P < 0.001) and an increased prevalence of advanced CKM (16.0 % vs. 9.4 %, P < 0.001). These associations were even more pronounced among individuals with NAFLD (moderate-to-severe SLD) or advanced liver fibrosis, who demonstrated greater metabolic dysregulation, systemic inflammation, and CKM burden (Table 2). Multivariable ordinal logistic regression demonstrated that NAFLD was independently associated with CKM stage progression (odds ratio [OR] = 2.01, 95 % CI: 1.84–2.20, P < 0.001) and higher CKD risk classification (OR = 1.29, 95 % CI: 1.09–1.52, P = 0.002) (Table 3). Sensitivity analyses using the mild-to-severe SLD definition of NAFLD produced consistent findings (Supplementary Table S5). Additionally, binary logistic regression showed that advanced CKM stages (vs. Stage 0) were associated with a nearly 3-fold increased risk of advanced liver fibrosis (OR = 2.96, 95 % CI: 1.23–7.13, P = 0.017), independent of socioeconomic factors (Supplementary Table S6).

**Table 2:** Baseline characteristics of the study population, stratified by NAFLD status.

Characteristic	NAFLD (Mild-Severe SLD)			NAFLD (Moderate-Severe SLD)			Advanced liver fibrosis		
	No N = 7095	Yes N = 3890	P-value	No N = 8539	Yes N = 2446	P-value	No N = 10524	Yes N = 461	P-value
Age, years	41.26 (0.44)	45.34 (0.41)	<0.001	41.44 (0.43)	47.27 (0.55)	<0.001	41.99 (0.39)	61.85 (0.69)	<0.001
Age group ( %)			<0.001			<0.001			<0.001
20-39	3583 (51.7)	1473 (39.5)		4244 (51.1)	812 (33.9)		5028 (49.1)	28 (5.1)	
40-59	2022 (32.7)	1341 (38.6)		2471 (33.1)	892 (40.6)		3271 (34.8)	92 (26.8)	
60-74	1490 (15.6)	1076 (21.8)		1824 (15.7)	742 (25.4)		2225 (16.1)	341 (68.1)	
Male ( %)	3215 (46.0)	1877 (51.3)	0.004	3853 (46.3)	1239 (53.6)	<0.001	4885 (47.7)	207 (47.8)	0.99
Race/ethnicity ( %)			<0.001			0.004			0.006
Mexican-American	1867 (4.7)	1463 (7.1)		2354 (4.9)	976 (7.8)		3232 (5.5)	98 (3.6)	
Non-Hispanic black	2102 (10.4)	883 (8.9)		2483 (10.3)	502 (8.4)		2827 (9.7)	158 (15.7)	
Non-Hispanic white	2822 (77.6)	1389 (74.9)		3337 (77.1)	874 (75.0)		4021 (76.8)	190 (73.9)	
Other	304 (7.3)	155 (9.2)		365 (7.7)	94 (8.8)		444 (8.0)	15 (6.8)	
Smoking status ( %)			<0.001			<0.001			<0.001
Never	3515 (45.6)	1895 (44.0)		4250 (46.0)	1160 (41.5)		5205 (45.2)	205 (42.7)	
Former	1543 (23.7)	1074 (31.0)		1883 (24.0)	734 (34.7)		2456 (25.7)	161 (38.4)	
Current	2037 (30.6)	921 (25.0)		2406 (30.1)	552 (23.7)		2863 (29.1)	95 (18.9)	
Less than high school ( %)	2334 (19.6)	1723 (27.9)	<0.001	2907 (20.3)	1150 (30.4)	<0.001	3809 (21.8)	248 (37.7)	<0.001
Married ( %)	4209 (63.8)	2469 (67.2)	0.024	5065 (63.5)	1613 (70.5)	<0.001	6410 (64.9)	268 (65.5)	0.87
Low income ( %)	2178 (17.1)	1328 (18.6)	0.3	2654 (17.1)	852 (19.8)	0.052	3337 (17.5)	169 (20.8)	0.2
Healthy eating ( %)	2272 (34.0)	1306 (35.3)	0.30	2760 (33.9)	818 (36.5)	0.15	3384 (34.1)	194 (44.5)	0.003
C-reactive protein, mg/dL	0.36 (0.01)	0.44 (0.01)	<0.001	0.37 (0.01)	0.49 (0.02)	<0.001	0.38 (0.01)	0.58 (0.06)	<0.001
Overweight / obeisty ( %)	3843 (48.2)	2925 (72.2)	<0.001	4753 (49.8)	2015 (81.7)	<0.001	6414 (55.3)	354 (77.1)	<0.001
Central obesity ( %)	2371 (28.0)	2315 (56.4)	<0.001	3048 (29.7)	1638 (68.0)	<0.001	4377 (36.3)	309 (65.4)	<0.001
Insulin resistance( %)									
HOMA-IR≥2.5	2136 (22.3)	2325 (51.6)	<0.001	2779 (23.9)	1682 (64.3)	<0.001	4171 (30.9)	290 (57.6)	<0.001
HOMA-IR≥2.0	3124 (35.5)	2776 (65.0)	<0.001	3953 (37.3)	1947 (77.2)	<0.001	5575 (44.4)	325 (65.3)	<0.001
HOMA-IR≥1.0	6312 (85.8)	3700 (93.9)	<0.001	7635 (86.3)	2377 (97.4)	<0.001	9584 (88.3)	428 (92.4)	0.04
Prediabetes ( %)	1926 (21.9)	1294 (30.9)	<0.001	2374 (22.9)	846 (32.7)	<0.001	3103 (24.8)	117 (23.16)	0.65
Diabetes ( %)	484 (4.32)	732 (13.8)	<0.001	650 (4.82)	566 (18.0)	<0.001	995 (6.31)	221 (42.6)	<0.001
Hypertension ( %)	3068 (39.5)	2256 (54.6)	<0.001	3754 (40.1)	1570 (62.0)	<0.001	4973 (43.4)	351 (74.9)	<0.001
High triglycerides ( %)	2126 (28.9)	2085 (54.1)	<0.001	2731 (30.4)	1480 (64.5)	<0.001	3984 (36.7)	227 (47.6)	0.003
MetS ( %)	1528 (18.1)	1940 (47.4)	<0.001	2012 (19.7)	1456 (60.4)	<0.001	3210 (26.7)	258 (56.1)	<0.001
CKD ( %)	1094 (12.7)	793 (18.1)	<0.001	1351 (13.0)	536 (20.1)	<0.001	1681 (13.5)	206 (45.5)	<0.001
CKD risk ( %)			<0.001			<0.001			<0.001
Low risk	5902 (86.6)	3035 (81.0)		7068 (86.2)	1869 (78.8)		8724 (85.9)	213 (49.1)	
Moderately increased risk	910 (11.0)	656 (15.4)		1129 (11.3)	437 (16.8)		1421 (11.7)	145 (34.5)	
High risk	184 (1.7)	137 (2.7)		222 (1.7)	99 (3.3)		260 (1.7)	61 (11.0)	
Very high risk	99 (0.7)	62 (1.0)		120 (0.7)	41 (1.0)		119 (0.6)	42 (5.3)	
CKM stage			<0.001			<0.001			<0.001
Stage 0	1366 (23.6)	388 (14.0)		1608 (23.5)	146 (7.8)		1,743 (21.0)	11 (3.3)	
Stage 1	1332 (17.7)	408 (9.8)		1,533 (17.0)	207 (7.5)		1724 (15.5)	16 (4.4)	
Stage 2	3514 (49.3)	2346 (60.1)		4287 (49.5)	1573 (66.4)		5656 (53.0)	204 (46.6)	
Advanced CKM	883 (9.4)	748.0 (16.0)		1111 (9.9)	520 (18.3)		1401 (10.5)	230 (45.7)	

Abbreviation: NAFLD, Nonalcoholic fatty liver disease; SLD, steatosis liver disease; CKD, chronic kidney disease; CKM, Cardiovascular-Kidney-Metabolic syndrome; CKD, chronic kidney disease; Continuous variables are presented as weighted means with standard errors, while categorical variables are presented as unweighted frequencies with weighted percentages.

**Table 3:** Ordered logistic regression of NAFLD association with progression of CKM and CKD risk.

Characteristic	CKM stage		CKD risk	
	OR(95 %CI)	P-value	OR(95 %CI)	P-value
NAFLD				
No	ref		ref	
Yes	2.01(1.84, 2.20)	<0.001	1.29(1.09, 1.52)	0.002
Age, years	1.08(1.08, 1.09)	<0.001	1.08(1.07, 1.09)	<0.001
Sex				
Female	ref		ref	
Male	1.86(1.66, 2.09)	<0.001	0.57(0.49, 0.67)	<0.001
Race/ethnicity		<0.001		
Mexican-American	ref		ref	
Non-Hispanic black	1.14(1.00, 1.30)	0.004	2.76(2.34, 3.26)	<0.001
Non-Hispanic white	0.82(0.71, 0.95)	0.01	1.24(1.00, 1.54)	0.052
Other	0.91(0.71, 1.18)	0.492	1.29(0.85, 1.95)	0.19
Married				
No	ref		ref	
Yes	0.95(0.84, 1.07)	0.413	0.95(0.79, 1.14)	0.56
Education level				
Less than high school	ref		ref	
High school graduate or higher	0.86(0.74, 1.01)	0.058	0.89(0.76, 1.04)	0.156
Smoking status				
Never	ref		ref	
Former	1.08(0.93, 1.25)	0.304	1.12(0.90, 1.39)	0.328
Current	1.29(1.12, 1.48)	<0.001	0.98(0.76, 1.26)	0.864
Poverty income rate	0.91(0.88, 0.95)	<0.001	0.97(0.93, 1.02)	0.209
Healthy eating index	1.00(0.99, 1.00)	0.11	1.00(0.99, 1.00)	0.337

Abbreviation: NAFLD, Nonalcoholic fatty liver disease; CKM, Cardiovascular-Kidney-Metabolic syndrome; CKD, chronic kidney disease; CKD risk categories are defined by the Kidney Disease Improving Global Outcomes (KDIGO) classification; OR, odds ratio; CI, confidence interval.

### 4.2 Association of NAFLD or liver fibrosis status with mortality

During a median follow-up of 27.9 years (95 % CI: 27.8–28.0), 3,772 all-cause deaths occurred, including 1,249 cardiovascular deaths. Age-standardized cumulative mortality rates varied significantly across NAFLD and CKM subgroups (Supplementary Table S7). NAFLD patients exhibited higher age-standardized all-cause mortality than non-NAFLD individuals (35.1 % vs. 29.4 %, P<0.001), with a pronounced gradient in advanced liver fibrosis (50.4 % vs. 30.1 %, P<0.001). Table 4 summarizes the relationship between NAFLD and all-cause as well as cardiovascular mortality. In both the univariate model (HR 1.78; 95 % CI: 1.59 - 1.99) and multivariate model 1 (HR 1.26; 95 % CI: 1.14 - 1.40), patients with NAFLD exhibited higher all-cause mortality compared to those without NAFLD. However, after adjusting for specific components of the CKM, the association between NAFLD and all-cause mortality was no longer significant. In contrast, the relationship between advanced liver fibrosis and all-cause mortality remained significant (HR 1.26; 95 % CI: 1.07 - 1.48).

**Table 4:** Association between NAFLD or liver fibrosis status and long-term mortality.

		Deaths, n	Univariable model		Multivariable model 1		Multivariable model 2	
			HR (95 % CI)	P-value	HR (95 % CI)	P-value	HR (95 % CI)	P-value
**All-cause mortality**								
NAFLD (Mild-Severe SLD)	No (n=7059)	2188	ref		ref		ref	
	Yes (n=3890)	1584	1.55 (1.44, 1.67)	<0.001	1.23 (1.13, 1.33)	<0.001	1.08 (0.98, 1.18)	0.10
NAFLD (Moderate-Severe SLD)	No (n=8539)	2673	ref		ref		ref	
	Yes (n=2449)	1099	1.78 (1.59, 1.99)	<0.001	1.26 (1.14, 1.40)	<0.001	1.07 (0.95, 1.20)	0.27
Advanced liver fibrosis	No (n=10524)	3389	ref		ref		ref	
	Yes (n=461)	383	5.28 (4.41, 6.31)	<0.001	1.49 (1.29, 1.71)	<0.001	1.26 (1.07, 1.48)	0.01
**Cardiovascular mortality**								
NAFLD (Mild-Severe SLD)	No (n=7059)	728	ref		ref		ref	
	Yes (n=3890)	521	1.52 (1.29, 1.78)	<0.001	1.15 (0.97, 1.35)	0.10	0.87 (0.73, 1.02)	0.09
NAFLD (Moderate-Severe SLD)	No (n=8539)	888	ref		ref		ref	
	Yes (n=2449)	361	1.75 (1.44, 2.13)	<0.001	1.17 (0.97, 1.39)	0.09	0.84 (0.68, 1.03)	0.09
Advanced liver fibrosis	No (n=10524)	1111	ref		ref		ref	
	Yes (n=461)	138	6.24 (4.86, 8.02)	<0.001	1.47 (1.16, 1.85)	0.001	1.10 (0.84, 1.44)	0.48

Multivariate model 1 was adjusted for age, sex, race, education level, marital status, smoking status, poverty income rate, and Healthy eating index.

Multivariate model 2 was adjusted for BMI, hypertension, diabetes, CKD risk, fasting triglycerides, HDL-C, and CRP in addition to model 1.

Abbreviation: NAFLD, non-alcoholic fatty liver disease; SLD, steatosis liver disease; HR, hazard ratio; CI, confidence interval; BMI, body mass index; CKD, chronic kidney disease; HDL-C, high-density lipoprotein cholesterol; CRP, C-reactive protein.

### 4.3 Comparison of mortality according to CKM status among subjects with NAFLD

**Fig. 2:**
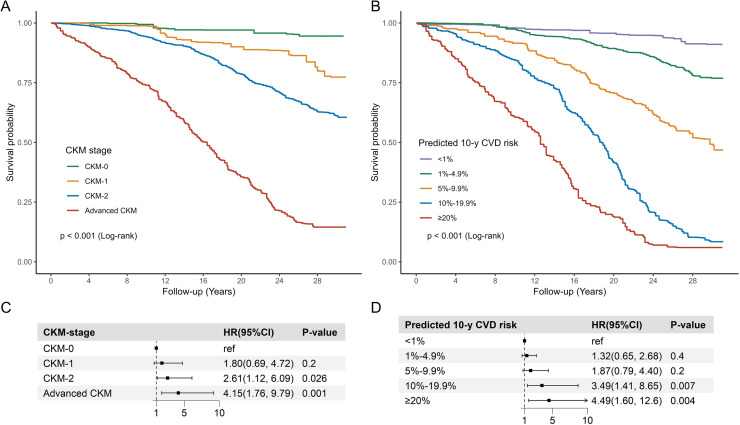
Survival analysis of the association between CKM and mortality among NAFLD subjects. Fig. legend: Kaplan-Meier survival curves for all-cause mortality in NAFLD patients stratified by CKM stage (**A**) and predicted 10-year CVD risk (**B**). Multivariable Cox regression analysis for the association of CKM stage (**C**) and predicted 10-year CVD risk (**D**) with all-cause mortality in NAFLD patients. The multivariable Cox regression models were adjusted for age, sex, race, income, marital status, education level, smoking status, poverty income rate, and healthy eating index. Abbreviation: CKM, Cardiovascular-Kidney-Metabolic syndrome; CVD, cardiovascular disease; HR, hazard ratio; CI, confidence interval; NAFLD, Nonalcoholic fatty liver disease.

**Fig. 3:**
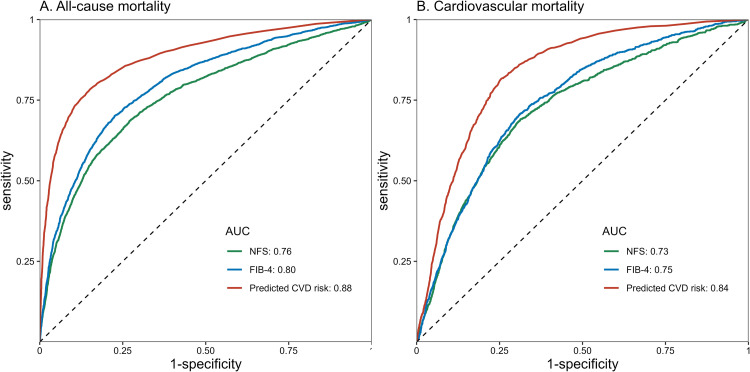
ROC curves of NFS, FIB-4, and predicted 10-year CVD risk in predicting mortality. Fig. legend: Predicted 10-year CVD risk was calculated by the full PREVENT equation model. Abbreviation: AUC, area under the curve; NFS, NAFLD fibrosis score; FIB-4, fibrosis-4 index; CVD, cardiovascular disease; ROC, receiver operating characteristic curve.

Among individuals with NAFLD, age-standardized all-cause mortality increased progressively with CKM severity, ranging from 23.0 % in Stage 0 to 34.1 % in Stage 2, peaking at 58.6 % in advanced CKM. A similar pattern was observed for cardiovascular mortality, with advanced CKM patients exhibiting a 3.8-fold higher risk compared to those in Stage 0 (20.6 % vs. 5.4 %, P < 0.001) (Supplementary Table S7). Kaplan-Meier curves revealed significant survival disparities across CKM stages (log-rank P < 0.001, Fig. 2A). After adjustment for socioeconomic factors, advanced CKM was associated with a 4.15-fold increased risk of all-cause mortality (HR = 4.15, 95 % CI: 1.76–9.79, P-value = 0.001) (Fig. 2C). Predicted 10-year CVD risk further stratified outcomes, with a ≥20 % risk linked to 4.49-fold higher mortality (HR = 4.49, 95 % CI: 1.60–12.6, P-value = 0.004) (Fig. 2D). These findings remained consistent in complete-case analyses, which further reinforced the robustness and internal validity of our results (Supplementary Fig. S2). Additionally, we performed a sensitivity analysis without merging CKM stages 3 and 4. The results showed a progressive increase in all-cause mortality risk across CKM stages, supporting the robustness of our main findings (Supplementary Table S8). For cardiovascular mortality, competing risk analyses accounting for non-CVD deaths revealed similar patterns. Cumulative incidence curves demonstrated clear and progressive stratification of cardiovascular mortality among individuals with NAFLD, both by CKM stage and predicted 10-year CVD risk categories, These findings underscore the prognostic utility of CKM staging and CVD risk estimation in this population (Gray’s test P < 0.001, Supplementary Fig. S3). Multivariable Fine-Gray models further confirmed the robustness of these associations (Supplementary Table S9-S10). Additionally, ROC analysis demonstrated that the PREVENT equation predicted 10-year CVD risk had superior prognostic value compared to the noninvasive fibrosis scores. As shown in Fig. 3, predicted 10-year CVD risk exhibited significantly higher predictive accuracy for all-cause and cardiovascular mortality (DeLong test, P < 0.001), with AUCs of 0.88 and 0.84, respectively.

### 4.4 Subgroup and mediation analysis

Supplementary Fig. S4 presents subgroup analysis of NAFLD with all-cause and cardiovascular mortality across different strata of age, sex, race, CKD risk, and CKM stage. No significant interaction effects were observed across these subgroups. Notably, the association between NAFLD and all-cause mortality weakened with increasing age, with a significant risk observed in younger adults (HR = 1.69, 95 % CI: 1.36–2.10 for ages 20–39) but diminishing in older age groups. Within the CKM subgroup, NAFLD was only significantly related to all-cause mortality in CKM Stage 2 (HR = 1.25, 95 % CI: 1.08–1.45), and no significant associations were observed in other CKM stages. For cardiovascular mortality, NAFLD was not significant in any subgroup.

**Fig. 4:**
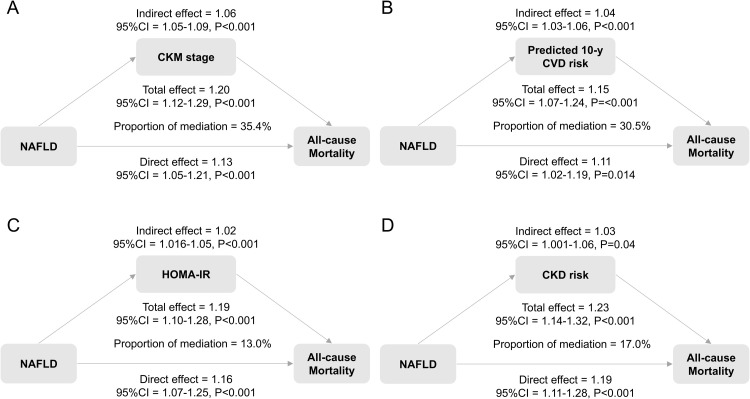
Mediation analysis of the association between NAFLD and all-cause mortality. Fig. legend: Models were adjusted for age, sex, race, income, marital status, education level, smoking status, poverty income rate, and healthy eating index. Abbreviation: NAFLD, Nonalcoholic fatty liver disease; CKM, Cardiovascular-Kidney-Metabolic syndrome; CVD, cardiovascular disease; HOMA-IR, homeostasis model assessment of insulin resistance; CKD, chronic kidney disease; CI, confidence interval.

Mediation analysis showed that the CKM stage significantly mediated the association between NAFLD and all-cause mortality. The total effect of NAFLD on all-cause mortality was 1.20 (95 % CI: 1.12–1.29, p < 0.001), with an indirect effect of 1.06 (95 % CI: 1.05–1.09, p < 0.001), accounting for 35.4 % of the total effect. Among the main CKM components, the mediation proportions were 30.5 % for predicted 10-year CVD risk, 13.0 % for HOMA-IR, and 17.0 % for CKD risk, respectively (Fig. 4). We also verified the mediating role of systemic inflammation, as indicated by CRP, mediating 5.8 % of the effect (Supplementary Table S11).

## 5 Discussion

By leveraging a large, nationally representative cohort of U.S. adults from NHANES III with extended mortality follow-up, this study is the first to systematically integrate NAFLD into the CKM syndrome framework. We comprehensively evaluated the relationship between NAFLD and CKM progression, as well as their associations with long-term all-cause and cardiovascular mortality. Importantly, we also delineated the prevalence patterns of NAFLD and advanced liver fibrosis across different CKM stages, providing new insights into the early hepatic involvement in the CKM continuum.

Previous research linking NAFLD, CKM syndrome, and mortality risk has largely focused on isolated components, such as MetS, type 2 diabetes, CKD, or CVD, without examining CKM as an integrated entity [[10], [13], [24]]. Our study addresses this critical gap by evaluating the role of NAFLD across the newly conceptualized CKM continuum and its association with long-term mortality. Notably, even among individuals classified as Stage 0, NAFLD remains prevalent, with an age-standardized prevalence of 8.4 % for NAFLD (moderate-to-severe SLD) and 21.8 % for NAFLD (mild-to-severe SLD). This observation suggests that NAFLD may act as an early driver of CKM progression rather than merely a downstream consequence of overt metabolic dysfunction. Emerging evidence supports this hypothesis, indicating that hepatic steatosis may precede systemic metabolic derangements, possibly mediated by subclinical insulin resistance or early adipose tissue dysfunction [25]. Genetic predispositions (e.g., PNPLA3 variants), high-calorie diets, physical inactivity, visceral fat accumulation, and gut dysbiosis may contribute to the development of ‘metabolically healthy or lean NAFLD’ [[26], [27], [28]]. As CKM advances, the prevalence of NAFLD rises markedly from 24.1 % at Stage 1 to 40.0 % at Stage 2, then stabilizes at 40.2 % in advanced CKM. In contrast, the prevalence of advanced liver fibrosis increases sharply in the advanced CKM stage, suggesting a shift from isolated metabolic dysfunction to multi-organ damage involving the heart, liver, and kidneys [29]. The evolving shift from NAFLD to MASLD introduces important implications for future research. Different diagnostic frameworks may identify distinct subtypes of steatotic liver disease, each with unique cardiometabolic risk profiles and prognostic trajectories within the CKM framework. Future studies are needed to clarify how these classification systems impact population-level estimates, risk stratification, and clinical decision-making across the spectrum of liver–cardiovascular–metabolic interactions.

Findings from previous cohort studies, including those based on NHANES III, have shown that while MAFLD remains independently associated with all-cause mortality after multivariable adjustment, NAFLD is not independently predictive of mortality [30,31]. Our results are consistent with these observations but extend them by using a longer follow-up and mediation analyses to explore underlying mechanisms. Our study demonstrates that NAFLD is associated with a 1.26-fold increase in all-cause mortality risk. However, after adjusting for specific CKM components, this association is no longer significant. Subgroup analyses further support this finding, showing that NAFLD is not independently associated with all-cause mortality when the CKM stage is accounted for. In the mediation analysis, CKM progression, CKD risk advancement, HOMA-IR, and CRP were all significant mediators of the association between NAFLD and all-cause mortality. These findings suggest that the increased mortality risk associated with NAFLD is primarily driven by CKM progression. Furthermore, our study underscores the utility of the CKM framework in refining NAFLD risk stratification beyond conventional approaches that rely on coexisting metabolic risk factors, noninvasive liver fibrosis scores (e.g., FIB-4, NFS), or liver stiffness measurements via vibration-controlled transient elastography [9]. Given that NAFLD significantly increases the risk of CKD, CVD, and type 2 diabetes, with cardiovascular death as the leading cause of mortality [32,33], a classification system based solely on liver fibrosis severity or metabolic dysfunction remains incomplete. Our findings demonstrate that CKM framework effectively stratifies overall mortality risk in NAFLD patients [20]. Notably, the PREVENT 10-year CVD risk score outperformed both the NFS and FIB-4 in predicting all-cause mortality among individuals with NAFLD. This finding highlights the dominant role of cardiovascular risk in shaping long-term prognosis in this population and supports the utility of cardiometabolic risk models in NAFLD/MASLD. However, it is important to note that NHANES III does not provide liver-specific mortality outcomes, and the relative performance of these scores in predicting liver-related deaths remains to be determined. Future research should evaluate and compare the predictive performance of these indicators and explore the development of an integrated risk assessment framework incorporating metabolic, cardiovascular, renal, and hepatic factors.

CKM syndrome is a complex, multisystem disorder driven by insulin resistance, dysfunctional adiposity, inflammation, and oxidative stress [2]. However, the current CKM framework seems insufficient to account for the liver’s central role in metabolic homeostasis and its intricate interactions with the cardiovascular and renal systems. As a key metabolic organ, the liver regulates lipid and glucose metabolism while actively contributing to CKM progression through hepatic insulin resistance, lipotoxicity, and systemic inflammation [29]. Hepatic insulin resistance, a hallmark of NAFLD, disrupts systemic metabolic balance by impairing glucose uptake and promoting hepatic gluconeogenesis, thereby exacerbating hyperglycemia and insulin demand, which accelerates the onset of diabetes and cardiovascular complications [34]. In parallel, the liver in NAFLD exhibits profound lipotoxicity and inflammation, releasing pro-inflammatory cytokines such as TNF-α and IL-6 and excess free fatty acids. These mediators amplify systemic inflammation and oxidative stress, creating a pro-atherogenic environment that fosters endothelial dysfunction, vascular remodeling, and an increased risk of cardiovascular events [35]. Furthermore, the liver and kidneys engage in bidirectional crosstalk, where hepatic dysfunction promotes renal injury through systemic inflammation and neurohormonal activation, while CKD exacerbates hepatic damage via uremic toxin accumulation and oxidative stress [24]. Additionally, the close interplay between the liver and heart creates a vicious cycle, where NAFLD-associated metabolic dysfunction and fluid overload worsen cardiac function, while heart failure leads to hepatic congestion and fibrosis, compounding disease progression. Given these interconnected pathophysiological mechanisms, future research and clinical strategies should move toward a more integrated model that recognizes NAFLD as a key determinant in CKM syndrome. Understanding the liver’s role as both a metabolic regulator and a mediator of systemic inflammation highlights the potential for targeted interventions in NAFLD to break the cycle of multi-organ dysfunction and improve clinical outcomes in CKM patients.

This study has several limitations. First, the use of NHANES III (1988–1994) limits the generalizability of our findings to contemporary populations. Over the past decades, the burden of metabolic abnormalities, such as NAFLD/MASLD, type 2 diabetes, obesity, and their cardiovascular consequences has continued to rise globally [36,37]. As a result, the burden of NAFLD/MASLD may be even greater in today's clinical and public health landscape. Future studies based on more recent cohorts with contemporary diagnostic approaches are needed to validate our findings and further explore the evolving relationship between steatotic liver disease and CKM health. Second, hepatic steatosis was assessed by ultrasound, while practical for large-scale surveys, but has limited sensitivity for detecting mild steatosis and may underestimate the true prevalence of NAFLD [38]. Third, NHANES III lacks documentation of several clinical cardiovascular conditions, such as atrial fibrillation and peripheral artery disease, which may have led to underestimation of clinical CVD (CKM stage 4) prevalence. Finally, the observed associations between NAFLD and CKM stages were derived from cross-sectional analysis, time series and causality could not be determined, and residual confounding may persist despite adjustments for various potential confounders.

## 6 Conclusions

This study highlights that CKM syndrome exhibits high comorbidity with NAFLD and serves as an effective framework for risk stratification in individuals with NAFLD. NAFLD is not an independent predictor of mortality but serves as a key driver to accelerate CKM progression to increase mortality. The high prevalence of hepatic steatosis in CKM Stage 0 emphasizes the existence of metabolically “silent” steatosis and underscores the importance of capturing early liver involvement before overt systemic dysfunction emerges.

## Consent for publication

Not applicable.

## Ethics approval and consent to participate

The study was considered exempt and approved by the institutional review board.

## Data availability

The National Health and Nutrition Examination Survey dataset are publicly available on the website (www.cdc.gov/nchs/nhanes/).

## Author agreement

We hereby confirm that this manuscript, titled "Prevalence and Long-Term Outcomes of NAFLD and Cardiovascular-Kidney-Metabolic Health in the United States", has not been published previously in any form, nor is it currently under consideration for publication elsewhere. All authors have thoroughly reviewed and approved the final version of the manuscript, and affirm that no conflicts of interest—financial, professional, or personal—exist that could influence the interpretation or reporting of this work.

## CRediT authorship contribution statement

**Jian Liu:** Writing – review & editing, Writing – original draft, Software, Methodology, Formal analysis, Conceptualization. **Lei Gao:** Writing – review & editing, Methodology, Funding acquisition. **Junlong Chen:** Validation, Methodology, Data curation. **Zhenghao Li:** Visualization, Validation, Methodology. **Wenhang Zhao:** Methodology, Investigation. **Shiwei Qin:** Validation, Methodology. **Xuesong Wen:** Writing – review & editing, Validation, Methodology. **Dongying Zhang:** Writing – review & editing, Supervision, Funding acquisition, Conceptualization.

## Declaration of competing interest

The authors declare that they have no known competing financial interests or personal relationships that could have appeared to influence the work reported in this paper.

## Appendix Supplementary materials

Image, application 1

Image, application 2
