# Supplementary material for: Prevalence and long-term outcomes of NAFLD and cardiovascular-kidney-metabolic health in the United States
Source: Am J Prev Cardiol. 2025 Jun 18;23:101049. doi: 10.1016/j.ajpc.2025.101049 (PMC12242466; doi:10.1016/j.ajpc.2025.101049)
Supplement: Supplementary file 1 [file mmc1.docx]

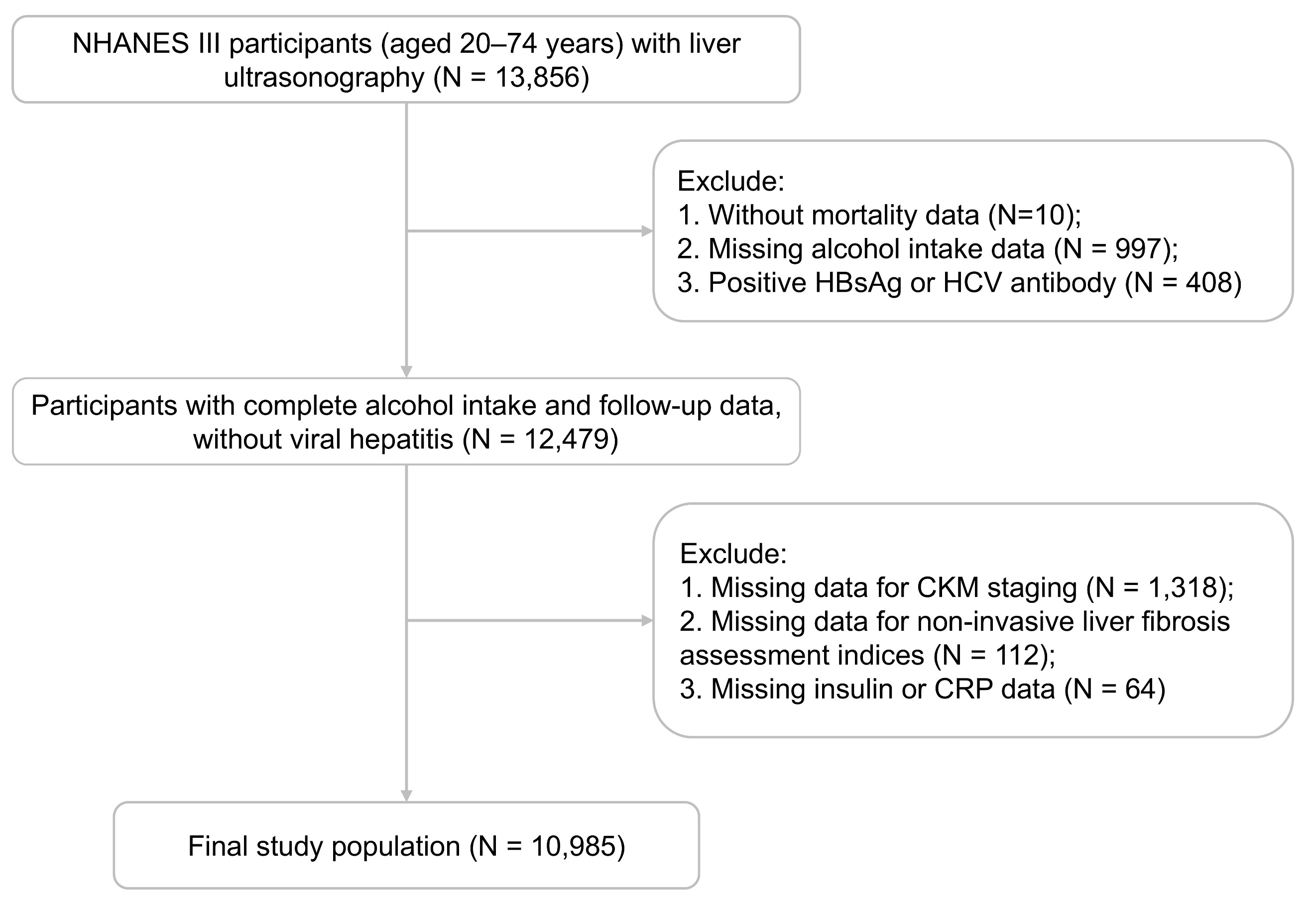


**Figure S1.** Flowchart of study participants.

Abbreviations: NHANES, National Health and Nutrition Examination Survey; CKM, Cardiovascular-Kidney-Metabolic syndrome; CRP, C-reactive protein.


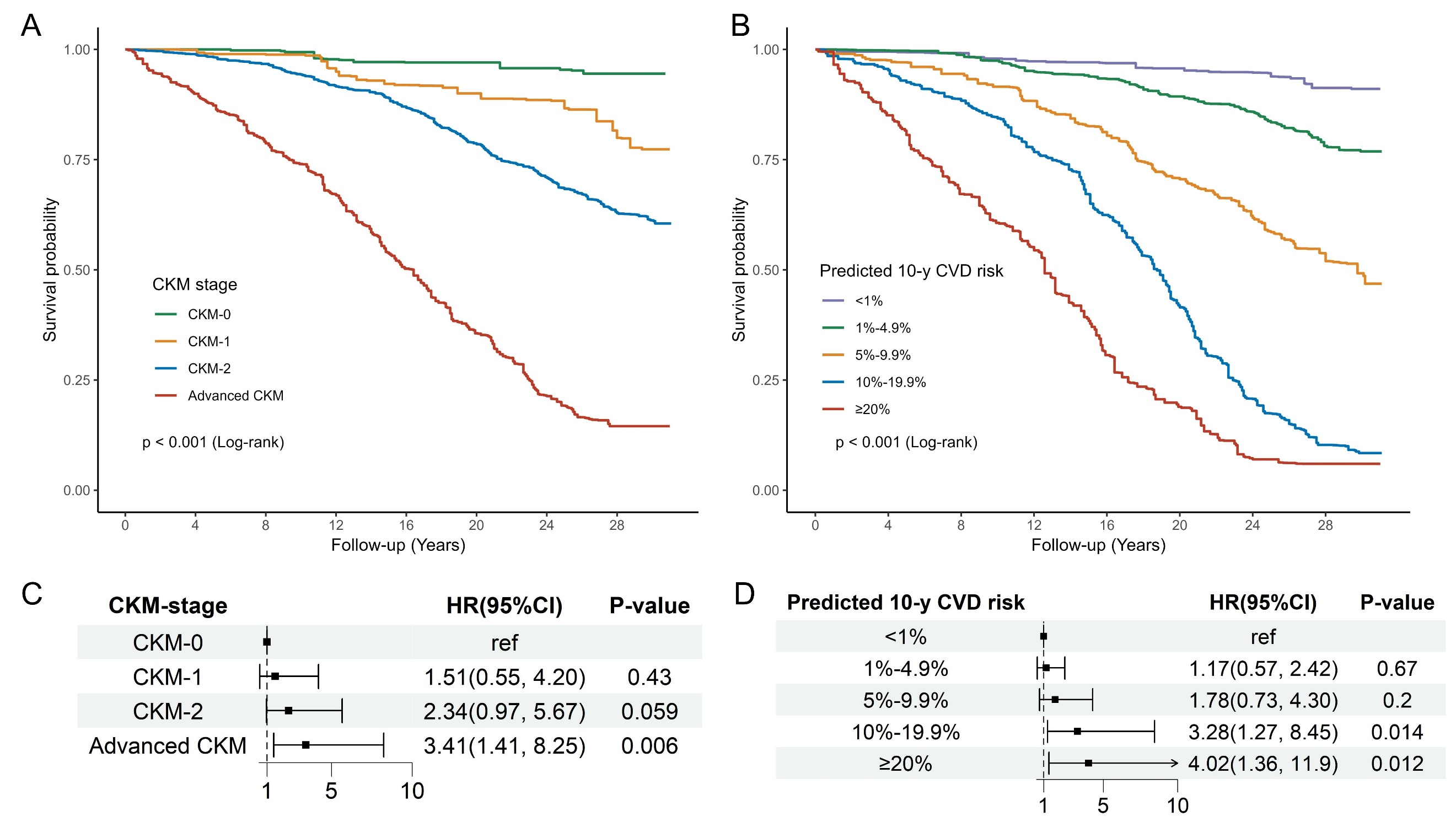


**Figure S2.** Survival analysis based on complete case analysis. Kaplan-Meier survival curves for all-cause mortality in NAFLD patients stratified by CKM stage (**A**) and predicted 10-year CVD risk (**B**). Multivariable Cox regression analysis for the association of CKM stage (**C**) and predicted 10-year CVD risk (**D**) with all-cause mortality in NAFLD patients.

The multivariable Cox regression models were adjusted for age, sex, race, income, marital status, education level, smoking status, poverty income rate, and healthy eating index.

Abbreviations: CKM, Cardiovascular-Kidney-Metabolic syndrome; CVD, cardiovascular disease; HR, hazard ratio; CI, confidence interval; NAFLD, Nonalcoholic fatty liver disease.


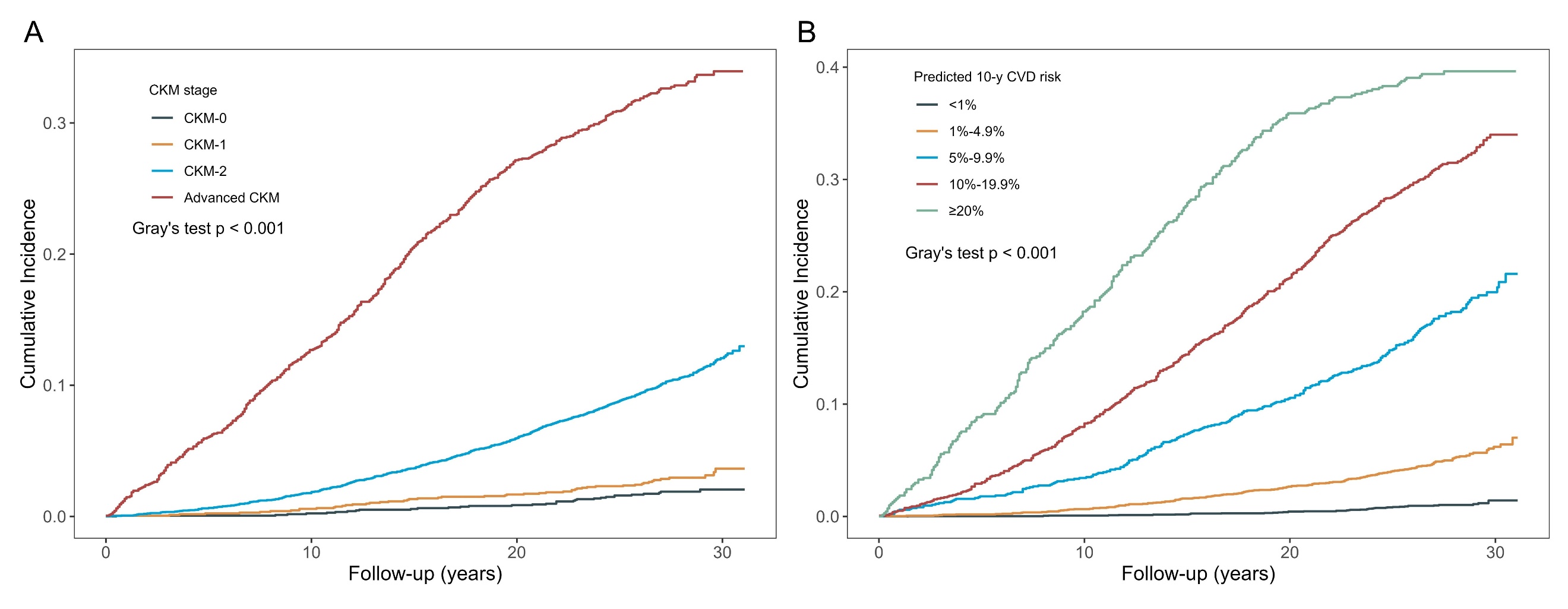


**Figure S3.** Cumulative incidence probability of cardiovascular mortality according to CKM stages (**A**) and predicted 10-year CVD risk (**B**) groups. Abbreviations: CKM, Cardiovascular-Kidney-Metabolic syndrome; CVD, cardiovascular disease.


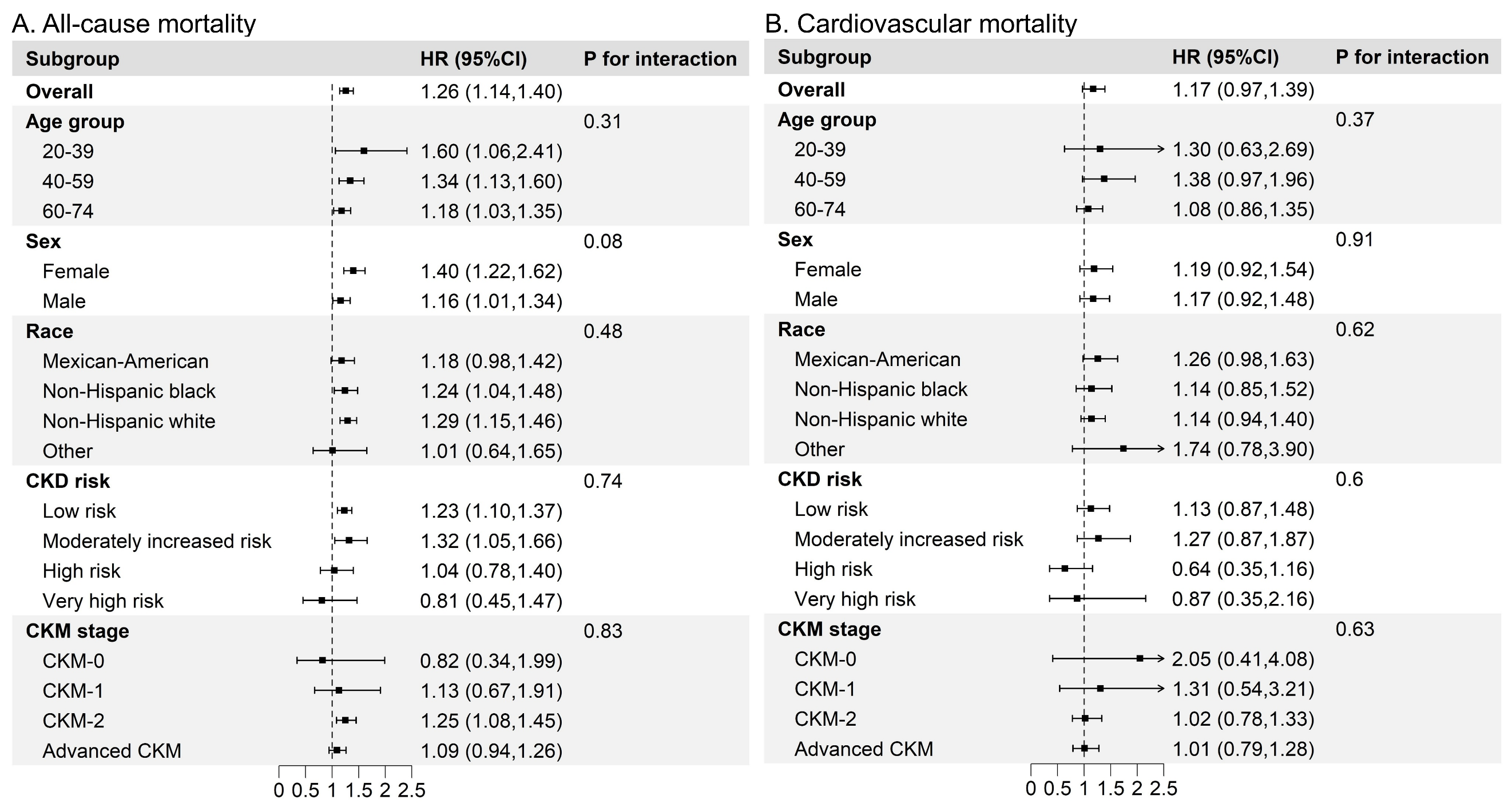


**Figure S4.** Subgroup analysis of the association between NAFLD and all-cause and cardiovascular mortality. Figure legend: All models were adjusted for age, sex, race, marital status, education level, smoking status, poverty income rate, and healthy eating index. Abbreviation: CKD, chronic kidney disease; CKM, Cardiovascular-Kidney-Metabolic syndrome; HR, hazard ratio; CI, confidence interval.
