# Supplementary material for: Prevalence and long-term outcomes of NAFLD and cardiovascular-kidney-metabolic health in the United States
Source: Am J Prev Cardiol. 2025 Jun 18;23:101049. doi: 10.1016/j.ajpc.2025.101049 (PMC12242466; doi:10.1016/j.ajpc.2025.101049)
Supplement: Supplementary file 2 [file mmc2.docx]

**Table S1.** Detailed definitions of Cardiovascular-Kidney-Metabolic (CKM) conditions.

| **CKM conditions** | **Definition** | **CKM indicators** | **Threshold for CKM indicators** |
| --- | --- | --- | --- |
| Metabolic disorders | Individuals with overweight/obesity, abdominal obesity, prediabetes, diabetes, hypertension, hypertriglyceridemia, or MetS. | Overweight/obesity | BMI ≥25 kg/m^2^ (or ≥23 kg/m^2^ if Asian ancestry) |
|  |  | Abdominal obesity | Waist circumference ≥88/102 cm in female/male (or if Asian ancestry  ≥80/90 cm in female/male) |
|  |  | Prediabetes | Fasting blood glucose ≥ 100-124 mg/dL or HbA1c ≥ 5.7%-6.4% and without self-reported diagnosis of diabetes, use of insulin, or oral hypoglycemic agents |
|  |  | Diabetes | Fasting blood glucose ≥ 125 mg/dL or HbA1c ≥ 6.5% or self-reported diagnosis of diabetes, use of insulin, or oral hypoglycemic agents |
|  |  | Hypertension | SBP ≥130 mmHg or DBP ≥80 mmHg or self-reported diagnosis of hypertension or use of antihypertensive medications |
|  |  | Hypertriglyceridemia | Triglycerides ≥ 135 mg/dL |
|  |  | MetS | MetS is defined by the presence of 3 or more of the following:   1. Waist circumference ≥88/102 cm in female/male (or if Asian ancestry ≥80/90 cm in female/male). 2. HDL cholesterol ≤ 50/40 mg/dL in female/male. 3. Triglycerides ≥150 mg/dL. 4. Elevated blood pressure (SBP ≥130 mmHg or DBP ≥80 mmHg and/or use of antihypertensive medications). 5. Fasting blood glucose ≥100 mg/dL |
| Kidney diseases | CKD risk by KDIGO classification | Low risk | eGFR ≥90 mL/min/1.73m² or eGFR 60-89 mL/min/1.73m², and UACR <30 mg/g |
|  |  | Moderately increased risk | eGFR 45-59 mL/min/1.73m² or eGFR 30-44 mL/min/1.73m² with UACR <30 mg/g; or eGFR ≥60 mL/min/1.73m² with UACR 30-299 mg/g |
|  |  | High risk | eGFR 30-44 mL/min/1.73m² with UACR 30-299 mg/g; or eGFR 45-59 mL/min/1.73m² with UACR ≥300 mg/g |
|  |  | Very high risk | eGFR <30 mL/min/1.73m²; or eGFR 30-44 mL/min/1.73m² with UACR ≥300 mg/g |
|  | Individuals with CKD | CKD | Moderate-to-high-risk CKD in KDIGO classification: UACR ≥ 30 mg/g  and eGFR ≥ 60 ml/min/1.73m^2^, UACR < 300 mg/g and eGFR ≤ 45-59  ml/min/1.73m^2^, or UACR < 30 mg/g and eGFR ≤ 30-44 ml/min/1.73m^2^. |
| CVD | Individuals with clinical CVD or subclinical CVD | Clinical CVD | History of chronic heart failure, coronary heart disease, heart attack, stroke, or electrocardiogram diagnosed myocardial infarction or atrial fibrillation. |
|  |  | Subclinical CVD | Any of the following criteria is met:   1. Very high-risk CKD in KDIGO classification: UACR ≥ 300 mg/g and eGFR ≤ 45-59 ml/min/1.73m^2^, UACR ≥ 30 mg/g and eGFR ≤ 30-44 ml/min/1.73m^2^, or eGFR ≤ 29 ml/min/1.73m^2^. 2. Predicted 10-year CVD risk ≥ 20% (≥ 20% using PREVENT [Predict Risk of cardiovascular disease EVENTs] full model). |

Abbreviations: BMI, body mass index; SBP, systolic blood pressure; DBP, diastolic blood pressure; MetS, metabolic syndrome; HDL, high-density lipoprotein; CKD, chronic kidney disease; KDIGO, The Kidney Disease Improving Global Outcomes; eGFR, estimated glomerular filtration rate; UACR, urinary albumin to creatinine ratio; CVD, cardiovascular disease.

Note: Asian was not listed as a separate race/ethnicity in NHANES III, therefore the uniform threshold for BMI and waist circumference was used in all participants.

**Table S2.** Definitions of CKM Syndrome Stages

| **CKM Syndrome Stage** | **Definition** |
| --- | --- |
| Stage 0: No CKM risk factors | Individuals with normal BMI and waist circumference, normoglycemia, normotension, a normal lipid profile, and no evidence of CKD or subclinical or clinical CVD |
| Stage 1: Excess or dysfunctional adiposity | Individuals with overweight/obesity, abdominal obesity, or prediabetes without the presence of other metabolic risk factors or CKD or CVD. |
| Stage 2: Metabolic risk factors and CKD | Individuals with metabolic risk factors (hypertriglyceridemia [≥135 mg/dL], hypertension, MetS, diabetes), or CKD without Subclinical/ Clinical CVD. |
| Stage 3: Subclinical CVD in CKM | Very high-risk CKD per KDIGO classification or High predicted 10-y CVD risk without clinical CVD. |
| Stage 4: Clinical CVD in CKM | Clinical CVD |

Abbreviations: CKM, Cardiovascular-Kidney-Metabolic syndrome; BMI, body mass index; CKD, chronic kidney disease; CVD, cardiovascular disease; KDIGO, The Kidney Disease Improving Global Outcomes.

**Table S3.** Baseline characteristics of eligible participants who had complete information

and who did not.

| **Characteristic** | **Total**  **(n = 10,985)** | **Completed**  **(n = 10,039)** | **Non-Completed**  **(n = 946)** | **P-value** |
| --- | --- | --- | --- | --- |
| Age, years (mean (SD)) | 44 (16) | 44 (16) | 45 (17) | 0.06 |
| Sex, n(%) |  |  |  | 0.6 |
| Female | 5,893 (54%) | 5,377 (54%) | 516 (55%) |  |
| Male | 5,092 (46%) | 4,662 (46%) | 430 (45%) |  |
| Race/ethnicity, n (%) |  |  |  | <0.001 |
| Mexican-American | 3,330 (30%) | 2,942 (29%) | 388 (41%) |  |
| Non-Hispanic black | 2,985 (27%) | 2,705 (27%) | 280 (30%) |  |
| Non-Hispanic white | 4,211 (38%) | 3,973 (40%) | 238 (25%) |  |
| Other | 459 (4.2%) | 419 (4.2%) | 40 (4.2%) |  |
| Smoking status, n(%) |  |  |  | 0.7 |
| Never | 5,410 (49%) | 4,933 (49%) | 477 (50%) |  |
| Former | 2,617 (24%) | 2,398 (24%) | 219 (23%) |  |
| Current | 2,958 (27%) | 2,708 (27%) | 250 (26%) |  |
| HEI(mean (SD)) | 63 (13) | 63 (13) | 63 (13) | 0.6 |
| PIR(mean (SD)) | 2.48 (1.79) | 2.48 (1.79) | 2.33 (1.53) | 0.9 |

Abbreviations: SD, standard deviation; HEI: healthy eating index; PIR, Poverty income rate.

**Table S4.** Baseline characteristics of participants who were included or exluded.

| **Characteristic** | **Overall**  N = 13,856 | **Included**  N = 10,985 | **Excluded**  N = 2,871 | **p-value**^1^ |
| --- | --- | --- | --- | --- |
| Age, Mean (SD) | 43.7 (16.0) | 43.8 (16.0) | 43.5 (16.0) | 0.13 |
| Sex, n (%) |  |  |  | 0.06 |
| Female | 7,377.0 (53.2%) | 5,893.0 (53.6%) | 1,484.0 (51.7%) |  |
| Male | 6,479.0 (46.8%) | 5,092.0 (46.4%) | 1,387.0 (48.3%) |  |
| Race, n (%) |  |  |  | <0.001 |
| Mexican-American | 4,159.0 (30.0%) | 3,330.0 (30.3%) | 829.0 (28.9%) |  |
| Non-Hispanic black | 4,080.0 (29.4%) | 2,985.0 (27.2%) | 1,095.0 (38.1%) |  |
| Non-Hispanic white | 5,036.0 (36.3%) | 4,211.0 (38.3%) | 825.0 (28.7%) |  |
| Other | 581.0 (4.19%) | 459.0 (4.18%) | 122.0 (4.25%) |  |
| Smoking status, n (%) |  |  |  | <0.001 |
| Current | 3,940.0 (28.4%) | 2,958.0 (26.9%) | 982.0 (34.2%) |  |
| Former | 3,181.0 (23.0%) | 2,617.0 (23.8%) | 564.0 (19.6%) |  |
| Never | 6,735.0 (48.6%) | 5,410.0 (49.2%) | 1,325.0 (46.2%) |  |
| HEI, Mean (SD) | 62.4 (13.2) | 63.0 (13.1) | 60.1 (13.4) | <0.001 |
| PIR, Mean (SD) | 2.5 (1.8) | 2.5 (1.8) | 2.2 (1.7) | 0.09 |
| Abbreviations: SD, standard deviation; HEI: healthy eating index; PIR, Poverty income rate. | | | | |

**Table S5.** Association of NAFLD (Mild-to-Severe SLD) with progression of CKM and CKD risk.

| **Characteristic** | **CKM stage** | | **CKD risk** | |
| --- | --- | --- | --- | --- |
|  | **OR(95%CI)** | **P-value** | **OR(95%CI)** | **P-value** |
| NAFLD |  |  |  |  |
| No | ref |  | ref |  |
| Yes | 1.60(1.45, 1.77) | <0.001 | 1.28(1.08, 1.52) | 0.01 |
| Age, years | 1.09(1.08, 1.09) | <0.001 | 1.08(1.07, 1.09) | <0.001 |
| Sex |  |  |  |  |
| Female | ref |  | ref |  |
| Male | 1.88(1.67, 2.10) | <0.001 | 0.57(0.49, 0.67) | <0.001 |
| Race/ethnicity |  | <0.001 |  |  |
| Mexican-American | ref |  | ref |  |
| Non-Hispanic black | 1.13(1.00, 1.27) | 0.05 | 2.78(2.37, 3.27) | <0.001 |
| Non-Hispanic white | 0.81(0.70, 0.94) | 0.01 | 1.25(1.00, 1.54) | 0.05 |
| Other | 0.89(0.69, 1.16) | 0.40 | 1.29(0.85, 1.95) | 0.23 |
| Married |  |  |  |  |
| No | ref |  | ref |  |
| Yes | 0.96(0.86, 1.09) | 0.55 | 0.95(0.79, 1.15) | 0.60 |
| Education level |  |  |  |  |
| Less than high school | ref |  | ref |  |
| High school graduate or higher | 0.86(0.74, 1.00) | 0.05 | 0.89(0.76, 1.04) | 0.15 |
| Smoking status |  |  |  |  |
| Never | ref |  | ref |  |
| Former | 1.09(0.94, 1.26) | 0.25 | 1.12(0.90, 1.39) | 0.32 |
| Current | 1.29(1.12, 1.49) | <0.001 | 0.98(0.76, 1.26) | 0.87 |
| Poverty income rate | 0.91(0.88, 0.95) | <0.001 | 0.97(0.93, 1.02) | 0.20 |
| Healthy eating index | 1.00(0.99, 1.00) | 0.11 | 1.00(0.99, 1.00) | 0.34 |

Abbreviation: NAFLD, Nonalcoholic fatty liver disease; SLD, steatosis liver disease; CKM, Cardiovascular-Kidney-Metabolic syndrome; CKD, chronic kidney disease; CKD risk categories are defined by the Kidney Disease Improving Global Outcomes (KDIGO) classification; OR, odds ratio; CI, confidence interval.

**Table S6.** Association between progression of CKM syndrome and advanced liver fibrosis

| **Characteristic** | **OR (95% CI)** | **P-value** |
| --- | --- | --- |
| CKM stage |  |  |
| Stage 0 | ref |  |
| Stage 1 | 1.06 (0.30, 3.76) | 0.93 |
| Stage 2 | 1.64 (0.76, 3.56) | 0.20 |
| Advanced CKM | 2.96 (1.23, 7.13) | 0.017 |
| Age, years | 1.09 (1.08, 1.11) | <0.001 |
| Sex |  |  |
| Female | ref |  |
| Male | 1.00 (0.61, 1.64) | 0.99 |
| Race/ethnicity |  |  |
| Mexican-American | ref |  |
| Non-Hispanic black | 1.70 (1.14, 2.53) | 0.011 |
| Non-Hispanic white | 0.80 (0.57, 1.13) | 0.20 |
| Other | 0.84 (0.35, 2.01) | 0.68 |
| Poverty income rate | 1.00 (0.90, 1.11) | 0.98 |
| Education level |  |  |
| Less than high school | ref |  |
| High school graduate or higher | 0.89 (0.60, 1.30) | 0.53 |
| Married |  |  |
| No | ref |  |
| Yes | 0.93 (0.60, 1.42) | 0.72 |
| Smoking status |  |  |
| Never | ref |  |
| Former | 0.90 (0.64, 1.26) | 0.52 |
| Current | 0.75 (0.46, 1.23) | 0.25 |
| Healthy eating index | 0.99 (0.98, 1.01) | 0.45 |

Abbreviation: CKM, Cardiovascular-Kidney-Metabolic syndrome; OR, odds ratio; CI, confidence interval.

**Table S7.** Age-standardized All-cause and Cardiovascular Mortality, Stratified by NAFLD status and CKM status among Individuals with NAFLD.

| **Group** | | **All-cause mortality (95% CI)** | **Cardiovascular mortality (95% CI)** |
| --- | --- | --- | --- |
| NAFLD (Mild-Severe SLD) | Yes | 33.1 (31.4, 34.9) | 10.5 (9.6, 11.6) |
|  | No | 29.3 (28.0, 30.6) | 9.4 (8.7, 10.1) |
| NAFLD (Moderate-Severe SLD) | Yes | 35.1 (32.9, 37.5) | 11.1 (9.9, 12.4) |
|  | No | 29.4 (28.2, 30.6) | 9.3 (8.7, 10.1) |
| Advanced liver fibrosis | Yes | 50.4 (40.4, 63.6) | 12.9 (8.8, 20.4) |
|  | No | 30.1 (29.0, 31.2) | 9.6 (9.0, 10.2) |
| CKM stage among NAFLD (Mild-Severe SLD) | Stage 0 | 21.4 (13.4, 33.0) | 4.9 (1.6, 12.1) |
|  | Stage 1 | 22.7 (17.2, 29.4) | 5.2 (2.8, 9.0) |
|  | Stage 2 | 32.8 (30.6, 35.1) | 9.6 (8.5, 10.9) |
|  | Advanced CKM | 57.1 (43.6, 75.6) | 21.3 (13.4, 34.7) |
| CKM stage among NAFLD (Moderate-Severe SLD) | Stage 0 | 23.0 (9.7, 47.5) | 5.4 (0.5, 23.4) |
|  | Stage 1 | 26.9 (18.6, 38.1) | 6.5 (2.8, 13.5) |
|  | Stage 2 | 34.1 (31.4, 37.0) | 10.0 (8.5, 11.6) |
|  | Advanced CKM | 58.6 (42.7, 81.9) | 20.6 (11.9, 37.5) |

Abbreviations: NAFLD, Nonalcoholic fatty liver disease; SLD, steatosis liver disease; CKM, Cardiovascular-Kidney-Metabolic syndrome; CI, confidence interval.

**Table S8.** Multivariable Cox regression analysis of the association between individual CKM stages (with CKM stages 3 and 4 separated) and all-cause mortality.

| **Characteristic** | **HR (95% CI)** | **P-value** |
| --- | --- | --- |
| CKM stage |  |  |
| Stage 0 | ref |  |
| Stage 1 | 1.50 (0.54 to 4.15) | 0.44 |
| Stage 2 | 2.31 (0.96 to 5.57) | 0.062 |
| Stage 3 | 2.77 (1.12 to 6.86) | 0.028 |
| Stage 4 | 3.61 (1.49 to 8.73) | 0.004 |
| Age, years | 1.09 (1.08 to 1.11) | <0.001 |
| Sex |  |  |
| Female | ref |  |
| Male | 1.10 (0.94 to 1.29) | 0.22 |
| Race/ethnicity |  |  |
| Mexican-American | ref |  |
| Non-Hispanic black | 1.43 (1.10 to 1.85) | 0.007 |
| Non-Hispanic white | 1.38 (1.10 to 1.74) | 0.006 |
| Other | 0.77 (0.52 to 1.13) | 0.18 |
| Poverty income rate | 0.96 (0.90 to 1.02) | 0.14 |
| Education level |  |  |
| Less than high school | ref |  |
| High school graduate or higher | 0.83 (0.66 to 1.05) | 0.13 |
| Married |  |  |
| No | ref |  |
| Yes | 0.87 (0.70 to 1.09) | 0.24 |
| Smoking status |  |  |
| Never | ref |  |
| Former | 1.16 (0.93 to 1.46) | 0.19 |
| Current | 2.16 (1.61 to 2.90) | <0.001 |
| Healthy eating index | 1.00 (0.99 to 1.00) | 0.71 |

Abbreviations: CKM, Cardiovascular-Kidney-Metabolic syndrome;

**Table S9.** Competing risk analysis of the association between CKM stages and cardiovascular mortality among Individuals with NAFLD.

| **Characteristic** | **OR (95% CI)** | **P-value** |
| --- | --- | --- |
| CKM stage |  |  |
| Stage 0 | ref |  |
| Stage 1 | 1.12 (0.73, 1.73) | 0.61 |
| Stage 2 | 2.30 (1.61, 3.30) | <0.001 |
| Advanced CKM | 3.44 (2.35, 5.03) | <0.001 |
| Age, years | 1.07 (1.06, 1.07) | <0.001 |
| Sex |  |  |
| Female | ref |  |
| Male | 1.16 (1.02, 1.32) | 0.02 |
| Race/ethnicity |  |  |
| Mexican-American | ref |  |
| Non-Hispanic black | 1.16 (0.83, 1.64) | 0.43 |
| Non-Hispanic white | 1.15 (0.85, 1.55) | 0.41 |
| Other | 0.96 (0.35, 2.01) | 0.68 |
| Poverty income rate | 0.90 (0.83, 0.98) | 0.02 |
| Education level |  |  |
| Less than high school | ref |  |
| High school graduate or higher | 1.01 (0.78, 1.33) | 0.93 |
| Married |  |  |
| No | ref |  |
| Yes | 0.95 (0.73, 1.24) | 0.71 |
| Smoking status |  |  |
| Never | ref |  |
| Former | 1.01 (0.77, 1.33) | 0.92 |
| Current | 1.29 (0.93, 1.77) | 0.12 |
| Healthy eating index | 1.00 (0.99, 1.01) | 0.91 |

Abbreviations: CKM, Cardiovascular-Kidney-Metabolic syndrome; NAFLD, Nonalcoholic fatty liver disease; OR, odds ratio; CI, confidence interval. HR, hazard ratio; CI, confidence interval.

**Table S10.** Competing risk analysis of the association between predicted 10-year CVD risk and cardiovascular mortality among Individuals with NAFLD.

| **Characteristic** | **HR (95% CI)** | **P-value** |
| --- | --- | --- |
| Predicted 10-y CVD risk* |  |  |
| <1% | ref |  |
| 1%-4.9% | 5.14 (2.03, 13.0) | <0.001 |
| 5%-9.9% | 9.14 (3.38, 24.8) | <0.001 |
| 10%-19.9% | 14.7 (5.23, 41.2) | <0.001 |
| ≥20% | 19.3 (6.52, 57.4) | <0.001 |
| Age, years | 1.02 (1.01, 1.04) | 0.01 |
| Sex |  |  |
| Female | ref |  |
| Male | 1.18 (0.93, 1.49) | 0.21 |
| Race/ethnicity |  |  |
| Mexican-American | ref |  |
| Non-Hispanic black | 1.14 (0.81, 1.59) | 0.51 |
| Non-Hispanic white | 1.21 (0.90, 1.62) | 0.23 |
| Other | 0.99 (0.52, 1.87) | 0.92 |
| Poverty income rate | 0.89 (0.81, 0.97) | 0.01 |
| Education level |  |  |
| Less than high school | ref |  |
| High school graduate or higher | 1.05 (0.81, 1.38) | 0.68 |
| Married |  |  |
| No | ref |  |
| Yes | 0.93 (0.71, 1.21) | 0.58 |
| Smoking status |  |  |
| Never | ref |  |
| Former | 1.00 (0.76, 1.32) | 0.94 |
| Current | 1.08 (0.78, 1.49) | 0.57 |
| Healthy eating index | 1.00 (0.99, 1.01) | 0.91 |

Abbreviations: CVD, cardiovascular disease; NAFLD, Nonalcoholic fatty liver disease; HR, hazard ratio; CI, confidence interval.

**Table S11.** Regression-based mediation analyses of the association between NAFLD and all-cause mortality.

| **Mediator** | **Total effect** | | **natural indirect effect** | | **natural direct effect** | | **Mediated proportion** | |
| --- | --- | --- | --- | --- | --- | --- | --- | --- |
|  | **HR (95%CI)** | **P-value** | **HR (95%CI)** | **P-value** | **HR (95%CI)** | **P-value** | **Proportion (95%CI)** | **P-value** |
| CKM stage | 1.20 (1.12, 1.29) | <0.001 | 1.06 (1.05, 1.09) | <0.001 | 1.13 (1.05, 1.21) | <0.001 | 35.4% (26.4%, 60.8%) | <0.001 |
| Predicted 10-y CVD risk | 1.15 (1.07, 1.24) | <0.001 | 1.04 (1.03, 1.06) | <0.001 | 1.11 (1.02, 1.19) | 0.014 | 30.5% (19.0%, 61.2%) | 0.002 |
| CKD-risk | 1.23 (1.14, 1.32) | <0.001 | 1.03 (1.00, 1.06) | 0.040 | 1.19 (1.11, 1.28) | <0.001 | 17.0% (0.6%, 29.4%) | 0.040 |
| HOMA-IR | 1.19 (1.10, 1.28) | <0.001 | 1.02 (1.02, 1.05) | <0.001 | 1.16 (1.07, 1.25) | <0.001 | 13.0% (8.8%, 36.4%) | <0.001 |
| C-reactive protein | 1.20 (1.11, 1.28) | <0.001 | 1.01 (1.00, 1.02) | <0.001 | 1.18 (1.10, 1.27) | <0.001 | 5.8% (2.8%, 13.0%) | <0.001 |

Models were adjusted for age, sex, race, income, marital status, education level, smoking status, poverty income rate, and healthy eating index.

Abbreviations: CKM, Cardiovascular-Kidney-Metabolic syndrome; CVD, cardiovascular disease; CKD, chronic kidney disease; HOMA-IR, Homeostatic Model Assessment - Insulin Resistance; OR, odds ratio; CI, confidence interval.
